# Management of Obstructive Sleep Apnea in an Edentulous Lower Jaw Patient with a Mandibular Advancement Device

**DOI:** 10.1155/2014/436904

**Published:** 2014-01-05

**Authors:** Filiz Keyf, Bülent Çiftci, Selma Fırat Güven

**Affiliations:** ^1^Department of Prosthodontics, Faculty of Dentistry, Hacettepe University, Ankara, Turkey; ^2^Sleep Disorders Center, Ataturk Chest Diseases and Thoracic Surgery Training and Research Hospital, Ankara, Turkey

## Abstract

Obstructive sleep apnea (OSA) is the most common sleep-related breathing disorder with periodic reduction or cessation of airflow during sleep. It is associated with loud snoring, disrupted sleep, and witnessed apneas. Treatment of OSA varies from simple measures such as oral appliances and nasal continuous positive airway pressure (CPAP) to surgical procedures like uvulopalatopharyngoplasty and tracheostomy. Oral appliances are a viable nonsurgical treatment alternative in patients with OSA, of which mandibular advancement devices are most common. Edentulism which contributes to the worsening of OSA reduces the number of available therapeutic strategies and is considered a contraindication to oral appliance therapy. This clinical report describes the treatment of a 63-year-old edentulous OSA patient for whom a mandibular advancement device was designed.

## 1. Introduction

Obstructive sleep apnea (OSA) is a common, chronic disorder of sleep and breathing that causes disability from pathologic sleepiness and respiratory and cardiovascular complications. The most common complaints are loud snoring and disrupted sleep. Of the many oral appliances designed for use in the treatment of OSA, mandibular advancement devices (MAD) have been the most intensely researched [1–5].

Although oral appliances can be used in a wide range of patients with OSA, they have several contraindications, one of which is the insufficient number of teeth in maxillary and mandibular arches.

A tooth is considered particularly important in obtaining retention and stability of the mandibular advancement device. Edentulism itself in turn contributes to the worsening of OSA and reduces the number of available treatment options. However, a search of the literature reveals only a few reports describing the treatment of edentulous patients with OSA using MAD [2–5].

The purpose of this case presentation is to report the use and results of a modified MAD as complete denture in an edentulous lower jaw patient with OSA.

## 2. Case Report

A 63-year-old woman was referred from the Council of the Sleep Related Breathing Disorders of Ataturk Chest Diseases and Chest Surgery Education and Research Hospital with a history and diagnosis of intrusive snoring and mild OSA. All participants underwent a full-night polysomnogram (PSG), using the compumedics voyager digital imaging 44-channel E-series system. Sleep stages as well as respiratory parameters were scored according to the standard criteria of American Academy of Sleep Medicine [6]. The patient was examined at the Department of Prosthodontics, Faculty of Dentistry, Hacettepe University. The patient had an apnea-hypopnea index (AHI) of 13.3 event/h and a minimum oxyhemoglobin saturation of 75% (Table 1). Patient complained of severe snoring, wake gasping and choking, daytime drowsiness, headache, and insomnia. The patient had snoring with the position only on back. Medical history of the patient did not reveal any preexisting diseases. History also revealed that patient did not have habit of sedatives, alcohol, or smoking.

**Figure 1:**
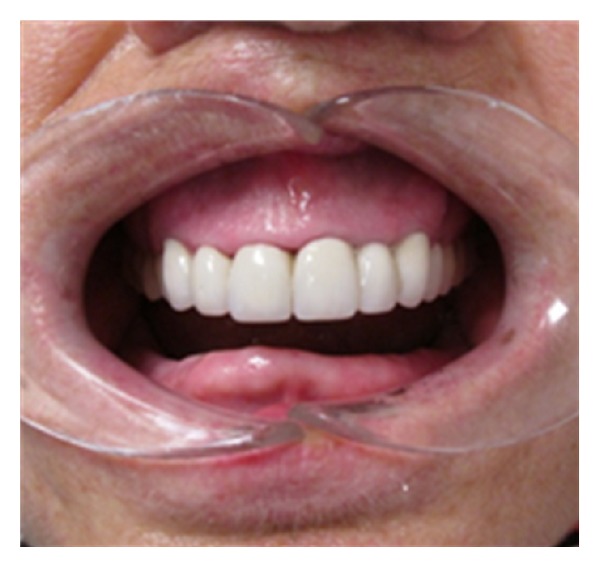
Intraoral view of the patient.

An intraoral examination of the patient was made, and intermaxillary relationship was evaluated. The patient had worn complete mandibular denture for many years and class I occlusion was observed (Figure 1). Palpation and auscultation were applied for temporomandibular joint (TMJ) evaluation. Muscle palpation and motion range of the jaw such as maximum opening (50 mm) and lateral and protrusive movements (>6 mm) were also evaluated. Neck size, obesity, oropharyngeal tissues, size of tongue, such as enlarged tongue, length of soft palate, and size of uvula, tonsils, and crowding of oropharyngeal area were other parameters of examination. Her body mass index (BMI) was 25.3 kg/m^2^.

**Figure 2:**
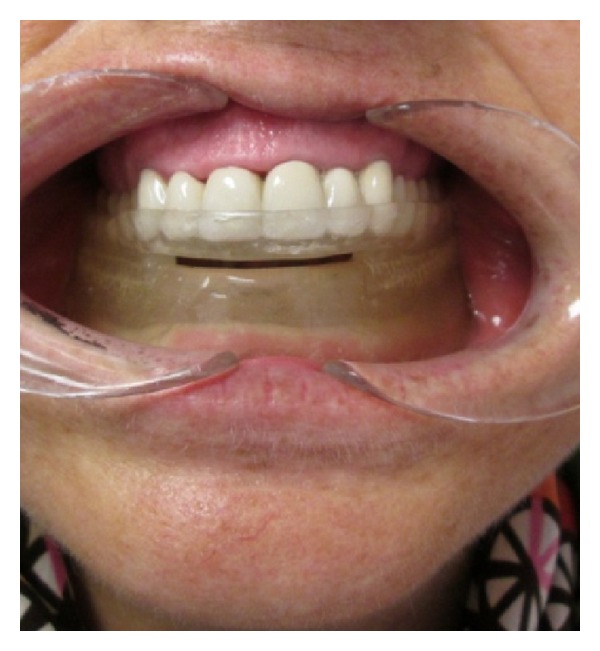
Device in patient's mouth.

**Figure 3:**
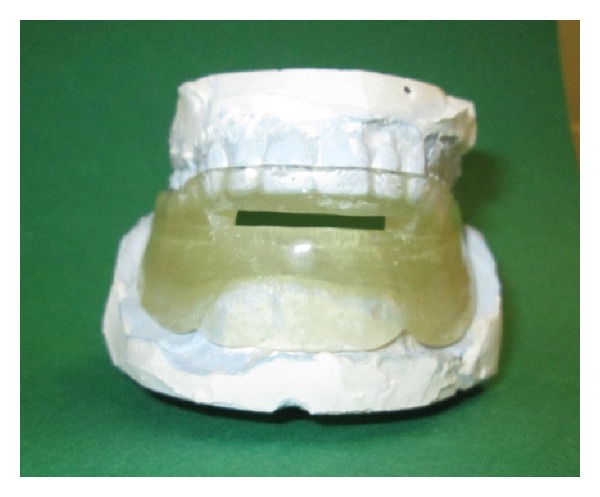
Device on the stone casts.

**Figure 4:**
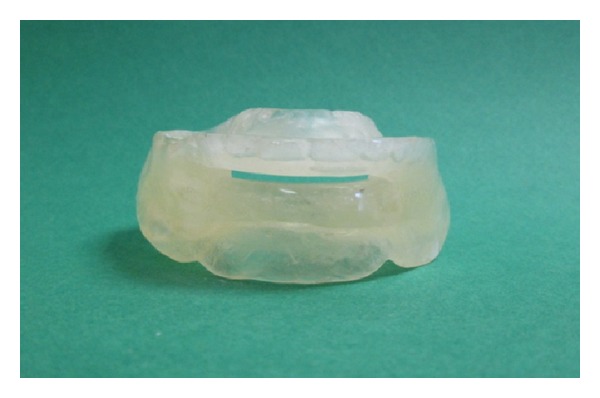
The appearance of MAD (out of the mouth).

Maxillary and mandibular preliminary impressions were made with irreversible hydrocolloidal impression material by using stock trays. The upper and lower casts were obtained. Custom trays were prepared for the lower jaw. Border molding the lower impression was made and functional impression was completed using zincoxide-eugenol impression pastes which were capable of recording soft tissues at rest. The hard stone cast was obtained. For recording the jaw relations, the shape of the edentulous dental arch using wax occlusal rim was prepared. The width and height of the occlusal table were adjusted. Maxillomandibular relation was determined to maintain the patient's rest vertical dimension. Centric relation position was marked on both of the wax rims bilaterally in the canine region. The patient was then asked to protrude maximally, and maximum protrusion position was marked bilaterally. The distance between the centric relation mark and the maximum protrusion mark on the maxillary rim was ascertained, and then 75% of the distance from the centric relation line was marked on the maxillary rim as the therapeutic position. The intermaxillary relations were recorded to fix the mandible at a protruded position with increased vertical dimension. The acrylic resin bases were prepared with heat-polymerized acrylic resin and the patient's vertical dimension of occlusion increased by 3 mm. A monoblock sleep device was prepared and delivered to the patient. Half palatal coverage was used, and retention of device was provided with undercuts of teeth. The device was evaluated in the patient's mouth for any discomfort (Figures 2, 3, and 4). The patient was able to breathe comfortably through the nasal airway. Instructions for use and care were provided at insertion of this appliance. The patient was advised to wear the appliance for at least 6 h during the night. The control PSG was performed at the fifth month of the treatment.

## 3. Results

During treatment, the patient reported a favorable sleeping pattern without any discomfort. Snoring, wake gasping, and choking were reduced drastically and she also reported improved sleep at night without apneas and her daytime headache and drowsiness had diminished considerably, which was also confirmed by the husband of the patient. After a followup period of five months, AHI was decreased from 13.3 to 3.0 with device. Significant decrease of AHI, shortening of apnea duration (from 31.5 sec to 16.5 sec), and changing in the oxygen desaturation index (from 16.0 to 3.4) were recognized during device use. During sleep, snoring did not occur and 22 respiratory events occurred; 3 of 22 respiratory events occurred as central apnea and hypopnea were found to be 19 of them. However, before treatment total number of respiratory event was 112 (Table 1).

## 4. Discussion

The literature revealed a fewer numbers of therapeutic strategies used in the treatment of edentulous patients with OSA [3–5, 7, 8]. This paper describes the clinical procedure of fabrication of a modified MAD for edentulous lower jaw patient with OSA and evaluates these results.

Intraoral appliances are generally advocated for mild OSA and simple snoring and in moderate to severe OSA as an alternative to nCPAP or craniofacial surgery. The use of intraoral appliances can be in form of soft palate lifting and tongue-retaining devices (TRD) [3, 4]. Among the entire range of appliances, MAD is the more commonly used and studied appliance. The primary mechanism of action of MAD is to cause mechanical advancement of the mandible and thereby increase the anteroposterior dimensions of the oropharynx. As this appliance requires dentitions for its retention it is generally contraindicated in edentulous patient; thus implant-retained MAD is the treatment of choice for edentulous OSA patients [4]. Implant-retained appliances are not a feasible treatment option in medically compromised patients, in patients who are economically poor, and in patients who lack motivation.

When reviewed through the literature, authors have proposed many modified techniques of fabricating the MAD for edentulous patient with OSA. Kurtulmus and Cotert [3] described a new functional splint combining a MAD and a TRD. Piskin et al. [5] reported modified MAD which displaces bulky masseter muscles laterally to provide more space for the tongue in edentulous patients. Arisaka et al. [7] indicated that just wearing the complete dentures during sleep improved the AHI in some edentulous OSA patients by restoring the vertical mandibular position without change in the horizontal mandibular position.

In this study, the wider the device base area is, the better the device retention will be. Therefore the functional impression making should be performed.

The device was fabricated with an advancement of 75% of maximum protrusion of the patient's maximum advancement of mandible in order to achieve maximum comfortable protrusion [9]. The amount of bite opening was adjusted to allow clearance of 3 mm between the upper and lower incisors during mandibular advancement, which would be sufficient enough to provide breathing space.

Although edentulousness is considered a contraindication for MAD, our patient reported no problems with the retention and stability of the appliance. Concerning sleep architecture, stage 1 percentage decreased. Sleep efficiency increased from 88.9% to 93.4%. Total REM sleep time increased from 84.5 min (16.9%) to 109.5 min (25.0%). AHI REM decreased from 34.1 to 2.2 and AHI non-REM from 9.1 to 3.3. Before treatment there were 14 obstructive apneas and 5 mixed apneas. During treatment they were zero. The number of central apnea was decreased from 9 to 3. Hypopnea decreased from 84 to 19. The average oxygen saturation did not improve as expected initially. It should be noted that the patient did not lose weight during this time. The patient indicated that her quality of life had improved and that she was comfortable with the device. These findings show that patient benefited from oral device.

## 5. Conclusion

This clinical report describes the technique of fabricating and results of oral device for an edentulous patient. The patient was satisfied with the modified device. The advantages of the device in study are that it is simple to fabricate, comfortable, and economical to the patient. Although oral appliances have been shown to be less effective than nasal CPAP therapy, these appliances should still be considered when patients are intolerant to CPAP treatment or had an unsuccessful surgery.

## Figures and Tables

**Table 1:** Polysomnographic parameters of the patient.

	Before treatment (without device) 09/19/2012	During treatment (with device) 02/21/2013
Total recording time (min)	561	469.7
Total sleep time (min)	499.0	438.5
Sleep latency (min)	5.0	11.0
REM latency (min)	124.5	72.5
Sleep efficiency (%)	88.9	93.4
Total REM sleep time (min)	84.5 (16.9%)	109.5 (25%)
Total stage N1 (min)	19.5 (3.9%)	11.0 (2.5%)
Total stage N2 (min)	239.5 (48.0%)	226.0 (51.5%)
Total stage N3 (min)	155.5 (31.2%)	92.0 (21.0)
AHI REM (events/hour)	34.1	2.2
AHI NREM (events/hour)	9.1	3.3
AHI (events/hour)	13.3	3.0
Obstructive apneas (number)	14	0
Mixed apneas (number)	5	0
Central apneas (number)	9	3
Hypopnea (number)	84	19
Longest duration of apnea (sec)	31.5	16.5
Total respiratory event (number)	112	22
Average SpO_2 _(%)	95	93
Sleep lowest SpO_2 _(%)	75	88
AHI supine (events/hour)	16.7	4.6
AHI right side (events/hour)	10.5	1.7
AHI left side (events/hour)	1.8	3.3
Awake average SpO_2 _(%)	95	94
Oxygen desaturation index	16.0	3.4

AHI: apnea-hypopnea index, SpO_2_: oxygen saturation by pulseoxymeter.
